# Mitochondrial Genomic Characteristics and Maternal Genetic Differentiation of Different Geographical Populations of *Saiga tatarica* in Kazakhstan

**DOI:** 10.3390/ani16142256

**Published:** 2026-07-21

**Authors:** Yue Pang, Zhumanov Kairat Toksanbaevich, Siyuan Wang, Nurpeisova Ainur Sultanovna, Bakirov Nurbol Zhumagadyrovish, Smagulov Darkhan Bakytbekovich, Wurelihazi Hazihan

**Affiliations:** 1 College of Animal Science and Technology, Shihezi University, Shihezi 832000, China; pangyue515@163.com (Y.P.); kairat_nur85@mail.ru (Z.K.T.); wangsiyuanme@126.com (S.W.); 2 Faculty of Veterinary Medicine, Kazakh National Agrarian Research University, Almaty 050010, Kazakhstan; nurbol979@mail.ru; 3 Kazakh Scientific Research Veterinary Institute, Almaty 050010, Kazakhstan; nurai1005@gmail.com; 4 Faculty of Agriculture and Veterinary Medicine, West Kazakhstan Innovation and Technology University, Uralsk 090009, Kazakhstan; dark.smagul@gmail.com

**Keywords:** *Saiga tatarica*, mitochondrial genome, genetic diversity, genetic differentiation

## Abstract

To assess the genetic diversity and matrilineal differentiation between the Betpak-Dala (BD) and Volga–Ural (VU) populations of *Saiga tatarica* in Kazakhstan, this study sequenced and comparatively analyzed the complete mitochondrial genomes of both populations. Phylogenetic analyses showed that *Saiga tatarica* clustered with the genera *Gazella*, *Nanger*, and *Eudorcas*, indicating a close evolutionary relationship. Genetic diversity analysis revealed that the BD population exhibited higher nucleotide diversity than the VU population. A total of 22 haplotypes were identified based on complete mitochondrial genome sequences, and no haplotypes were shared between the two populations, indicating pronounced matrilineal genetic differentiation. Synonymous mutations predominated among protein-coding genes, with the NADH dehydrogenase subunit 2 gene (*nd2*) and NADH dehydrogenase subunit 5 gene (*nd5*) genes exhibiting high levels of variation and serving as key regions for the accumulation of genetic diversity within the mitochondrial genome. These findings enhance the understanding of the genetic diversity and matrilineal genetic structure of *Saiga tatarica* populations in Kazakhstan and provide valuable information for their conservation and management.

## Simple Summary

The *Saiga tatarica* is an ancient species of the Eurasian steppe ecosystem, whose population once plummeted due to overhunting and disease outbreaks. To assess the genetic diversity of different geographic populations, this study analyzed the complete mitochondrial genomes of two populations, Betpak-Dala (BD) and Volga–Ural (VU). The results revealed that the BD population exhibited higher genetic diversity than the VU population. No shared haplotypes were identified between the two populations, suggesting a degree of maternal genetic differentiation. Furthermore, the mitochondrial control region (D-loop), NADH dehydrogenase subunit 2 gene (*nd2*) and NADH dehydrogenase subunit 5 gene (*nd5*) regions exhibited relatively high levels of genetic variation. This study revealed the genetic structure of different *Saiga tatarica* populations and provides a basis for their conservation and management.

## 1. Introduction

The saiga antelope (*Saiga tatarica*) belongs to the Artiodactyla order, the Bovidae family, and the Antilopinae subfamily. It is one of the oldest extant ungulate herbivores in the Eurasian steppe ecosystem [1], currently distributed primarily across Kazakhstan, Russia, and Mongolia, with a small captive population also maintained in Gansu Province, China [2,3]. Thanks to the intensive conservation efforts of the international community, the global population of saiga antelopes has increased significantly over the past decade. In 2023, the International Union for Conservation of Nature (IUCN) downgraded the species from Critically Endangered (CR) to Near Threatened (NT) [4]. Nevertheless, the species remains protected under Appendix II of the Convention on the Conservation of Migratory Species of Wild Animals (CMS) and Appendix II of the Convention on International Trade in Endangered Species of Wild Fauna and Flora (CITES), and is designated as a Class I National Key Protected Wild Animal in China [5]. Despite recent population recovery, the long-term viability of wild populations remains threatened by multiple factors, including habitat fragmentation, infectious disease outbreaks, poaching, and barriers to seasonal migration.

Based on geographic distribution, saiga antelopes are divided into several populations, including the Northwest Pre-Caspian Population (NWP), Volga–Ural (VU), Ustyurt (U), Betpak-Dala (BD), and the Mongolian saiga population [6]. Among them, the VU and BD populations are primarily distributed in Kazakhstan and are classified as *Saiga tatarica tatarica* (*S. t. tatarica*). The two populations differ in habitat characteristics, geographic distribution, and the intensity of conservation management. The VU population is distributed in northwest Kazakhstan, mainly inhabiting semi-desert and shrub areas. Its distribution range is relatively concentrated, and conservation management is comparatively well established. In contrast, the BD population is distributed in a vast area in central Kazakhstan, and its habitat types are mainly grassland and small hilly areas. The population distribution is more scattered and the environmental composition is more diverse [4].

Over the past century, saiga antelopes has experienced at least two major population bottlenecks. As populations declined, the geographic ranges of various local populations contracted markedly, and both the distances of seasonal migrations and the proportion of individuals participating in these migrations decreased significantly. The distribution ranges of the BD and VU populations were reduced to approximately 30% and 41% of their historical maximum extents, while migration distances declined by approximately 50% and 68%, respectively [7]. Currently, with ongoing population recovery, the distribution ranges of both the VU and BD populations are gradually expanding, and they have become the two principal recovering populations in Kazakhstan [2]. Although the population has shown signs of recovery in recent years, previous studies have indicated that severe population declines and restricted migratory movements may have reduced gene flow among populations, exacerbated genetic drift and inbreeding, and consequently diminished genetic diversity and the long-term adaptive potential of the species [8]. Therefore, assessing genetic variation and population differentiation in recovering populations is essential for elucidating recovery mechanisms and developing effective conservation strategies.

At present, research on the saiga antelope has mainly focused on population dynamics, migration ecology, and conservation management. Genetic diversity and population structure of different geographical populations of the saiga antelope were evaluated in previous studies based on analyses of the mitochondrial control region (D-loop), partial mitochondrial gene fragments, microsatellite markers, and k-mer data. Generally, the genetic differentiation among existing saiga antelope populations is relatively weak, but there are still differences in haplotype composition and genetic diversity levels among different geographical populations [2,6,9]. Among them, the Mongolian subspecies exhibits unique maternal lineage characteristics, and its genetic diversity is lower than that of the nominate subspecies [9]. Ancient DNA research further shows that the mitochondrial genetic diversity of modern saiga antelopes has significantly decreased compared with that during the Pleistocene era, indicating that they have experienced genetic diversity loss and population bottlenecks during their long-term evolutionary history [10].

With the development of high-throughput sequencing technologies, complete mitochondrial genomes can provide more comprehensive genetic information than traditional mitochondrial fragments and have become a powerful tool for elucidating population genetic structure and phylogenetic relationships [11]. However, comparative analyses of complete mitochondrial genomes between the two major recovering populations in Kazakhstan—the VU and BD populations—remain limited. Therefore, this study conducted comparative analyses of complete mitochondrial genome sequences from the BD and VU recovering populations in Kazakhstan to assess their genetic diversity, haplotype composition, and population differentiation, with the aim of elucidating genetic structure variation among geographically distinct populations and providing a genetic basis for the conservation and management of *Saiga tatarica*.

## 2. Materials and Methods

### 2.1. Sample Collection and Genomic DNA Extraction

Muscle tissues were collected from 15 individuals of the northwestern VU population and 15 individuals of the central BD population of *Saiga tatarica* in Kazakhstan. Samples were collected randomly from each population. Total genomic DNA was extracted using a genomic DNA extraction kit (DP304, Tiangen, Beijing, China). The concentration and purity of the extracted DNA were assessed to confirm that they met the quality standards required for subsequent complete mitochondrial genome sequencing.

### 2.2. Mitochondrial Genome Resequencing, Assembly, and Annotation

Complete mitochondrial genomes of 30 saiga antelopes were sequenced from total genomic DNA by Novogene (Beijing, China) on a NovaSeq 6000 platform (Illumina, San Diego, CA, USA) using a paired-end library protocol. The average insert size was 500 bp, and the resulting reads were 2 × 150 bp in length. Raw reads were processed with Trimmomatic v0.36 for adapter trimming and quality filtering, and the resulting clean reads were retained for subsequent analyses. De novo assembly of the mitochondrial genome for each individual was performed using Geneious Prime v2025.0.2 [12]. The assembled mitochondrial genomes were further checked by comparison with the reference mitochondrial genome and by confirming the completeness of mitochondrial gene annotation. Mitochondrial genome annotation was carried out using Geneious Prime and the MITOS Web Server [13].

### 2.3. Single Nucleotide Polymorphism (SNP) Detection

Multiple sequence alignment was performed using the MUSCLE algorithm in MEGA v12.0.11 [14], and the precise positions of the start codons and stop codons for each mitochondrial genome feature were further refined. When the annotation pipeline identified a noncanonical start codon or an incomplete stop codon, the boundaries of the corresponding gene were determined by aligning it with its counterpart in the reference genome (accession number: NC_020746.1) [15]. MEGA was used to calculate the number of variable sites for each gene in each sample. Based on the resulting variable site matrix, a heatmap was generated using the Weishengxin website (https://www.bioinformatics.com.cn/, accessed on 15 May 2026). 

### 2.4. Haplotype Network

The mitochondrial genome-wide haplotype diversity (*Hd*) was analyzed using DNAsp v6.12.03 (parameters: datatype = dna; Genome = Haploid; Chromosomal Location = mitochondrial) [16], and a TCS haplotype network was subsequently constructed in PopART v1.7 to visualize the relationships among haplotypes [17].

### 2.5. Phylogenetic Tree Construction

To clarify the phylogenetic position of the samples in this study within the Bovidae, we selected representative species of the Antilopinae as the ingroup and included the genus Oryx from the Hippotraginae, as well as the genera *Ovis aries*, *Capra hircus*, and *Pantholops hodgsonii* from the Caprinae, as comparative outgroups. *Bos taurus*, *Rangifer tarandus*, and *Alces alces* were used as the outgroup. Complete mitochondrial genome sequences of all comparative taxa were downloaded from the NCBI GenBank database. Detailed species information and GenBank accession numbers are provided in Table S1. Phylogenetic analyses were conducted using both the maximum likelihood (ML) and Bayesian inference (BI) methods, based on a complete mitochondrial genome dataset. ML analysis was performed using IQ-TREE v2.2.2.6 [18], with the ModelFinder module’s -m MFP option employed to automatically select the optimal nucleotide substitution model. Node support was assessed via 1000 ultrafast bootstrap replicates and 1000 SH-aLRT replicates, with parameter settings of --alrt 1000 -B 1000 and thread allocation set to automatic detection (-T AUTO).

Bayesian inference (BI) was performed using MrBayes v3.2.7 [19], with the nucleotide substitution model set to GTR+G. Markov chain Monte Carlo (MCMC) sampling was run for 1,000,000 generations, sampling every 1000 generations, the first 25% of the samples were discarded as burn-in. When the average standard deviation of split frequencies (ASDSF) was below 0.01 and the PSRF approached 1.00, the analysis was considered to have reached convergence. The final phylogenetic tree was visualized using iTOL v7 [20].

### 2.6. Analysis of Mitochondrial Genome Structure and Genetic Diversity

DnaSP was used to analyze the genetic diversity and population genetic structure of the mitochondrial genomes of saiga antelopes from the two populations. The main statistical indices included the number of segregating sites (*S*), number of polymorphic sites, nucleotide diversity (*π*), average number of nucleotide differences (*k*), Fixation Index (*Fst*), gene flow (*Nm*), Tajima’s D and Fu’s Fs neutrality test parameters, and polymorphic sites and shared mutation sites.

Relative synonymous codon usage (RSCU) values of the 13 mitochondrial protein-coding genes were calculated and visualized in R v4.5.0 using an R script obtained from BioDataTools v2.39. The input file consisted of concatenated sequences of the 13 mitochondrial protein-coding genes, and codon assignment was performed using the vertebrate mitochondrial genetic code (codon table = 2). The resulting RSCU values were presented as bar charts. The mitochondrial genome map was drawn using the OGDraw tool in the GeSeq platform, and was further refined and arranged using Adobe Illustrator v27.0. Finally, we used the OGDRAW module in the GeSeq website (https://chlorobox.mpimp-golm.mpg.de/geseq.html, accessed on 17 May 2026) to draw the mitochondrial genome map.

## 3. Results

### 3.1. Mitochondrial Genome Structure and Base Composition

Sequencing data of the 30 samples showed that after filtering, the clean data ranged from 6.06 to 6.62 Gb, the error rate was 0.01%, Q20 > 99.5%, and Q30 > 97%. The data quality was good and met the requirements for subsequent assembly and analysis (Table S2).

**Figure 1:**
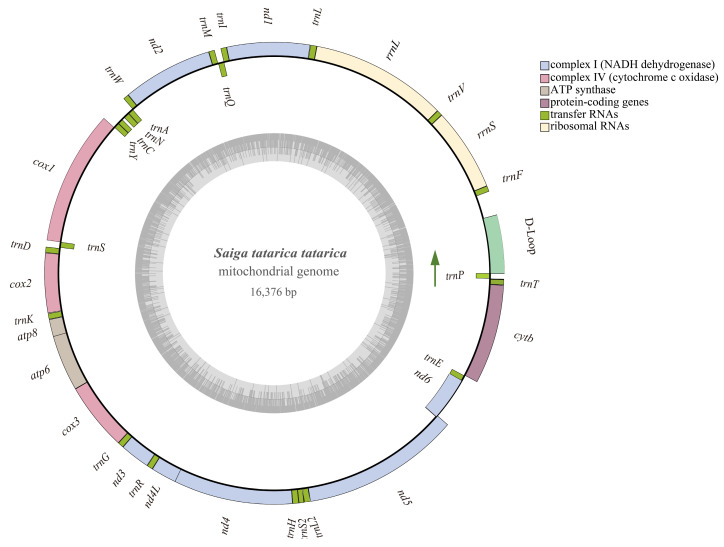
Complete mitochondrial genome organization of *Saiga tatarica* in Kazakhstan (16,376 bp). The genome consists of 13 protein-coding genes, 22 tRNA genes, two ribosomal RNA genes and a D-loop. Gene orientations are indicated by arrow direction. The mitogenome represents overlapping data from thirty antelopes.

The mitogenome of the saiga antelope from Kazakhstan is 16,376 bp in length, containing 37 genes, including 13 protein-coding genes (PCGs), 22 transfer RNA (tRNA) genes and 2 ribosomal RNA (rRNA) genes (Figure 1). The 13 PCGs comprise three cytochrome c oxidase subunit genes (*cox1*–*cox3*), one cytochrome b gene (*cytb*), seven NADH dehydrogenase subunit genes (*nd1*–*nd6* and *nd4L*), and two ATP synthase subunit genes (*atp6* and *atp8*). The two rRNA genes, *rrnL* and *rrnS*, encode the large and small ribosomal RNA subunits, respectively. The 13 PCGs total 11,406 bp in length, with gene sizes ranging from 201 bp (*atp8*) to 1,821 bp (*nd5*), and together encode 3,801 amino acids. Among the 13 PCGs, 10 genes (*cox1*–*cox3*, *atp8*, *atp6*, *nd1*, *nd4*, *nd4L*, *nd6*, and *cytb*) used ATG as the start codon, while three genes (*nd2*, *nd3*, and *nd5*) used ATA as the start codon. Regarding stop codons, eight genes (*cox1*, *cox2*, *atp8*, *atp6*, *nd4L*, *nd5*, *nd6*, and *cytb*) used TAA as the stop codon, four genes (*nd2*–*nd4*, *cox3*) ended with an incomplete stop codon (T), and *nd1* ended with an incomplete stop codon (TA) (Table 1).

**Figure 2:**
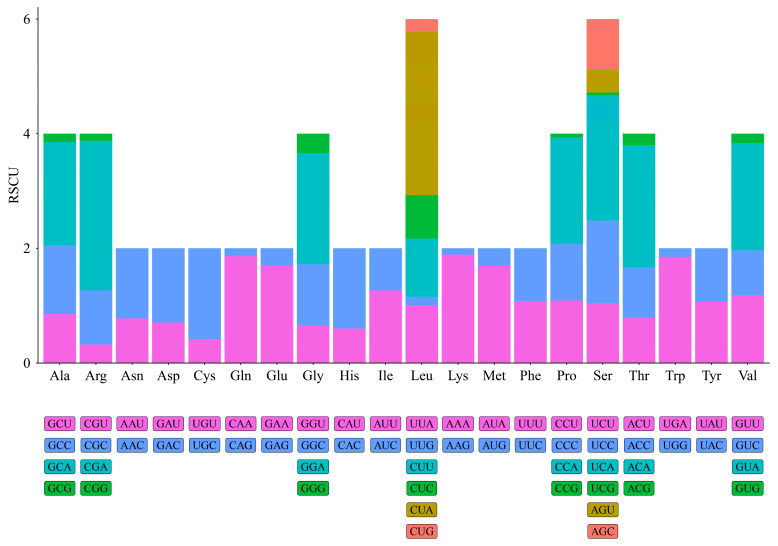
Relative synonymous codon usage (RSCU) of *Saiga tatarica* in Kazakhstan.

The relative synonymous codon usage (RSCU) showed the relative synonymous codon usage frequency of the 13 mitochondrial protein coding genes in the 30 samples (RSCU). The results show that the most frequently used codons include UUA (Leu), UCU (Ser), AUU (Ile), and GCU (Ala). These preferred codons mostly ended with A or U, indicating that the codon usage pattern of this mitogenome has a clear A/T bias. Comparing the VU and BD populations separately, we found that the codon usage patterns of mitochondrial protein coding genes were broadly similar in both groups, with no pronounced population specific codon biases observed. Accordingly, the overall RSCU values from the 30 samples were used to present the mitochondrial codon usage characteristics (Figure 2). Further comparison of base composition between the two geographical populations revealed that in the VU population, the average contents of A, T, C, and G were 33.84%, 28.37%, 24.78%, and 13.00%, respectively, while in the BD population, they were 33.86%, 28.33%, 24.81%, and 13.00%, respectively. The mitochondrial genomes of the two populations exhibit only minor differences in base composition, both showing a high AT content, indicating that the overall nucleotide composition is relatively conserved among the populations.

**Figure 3:**
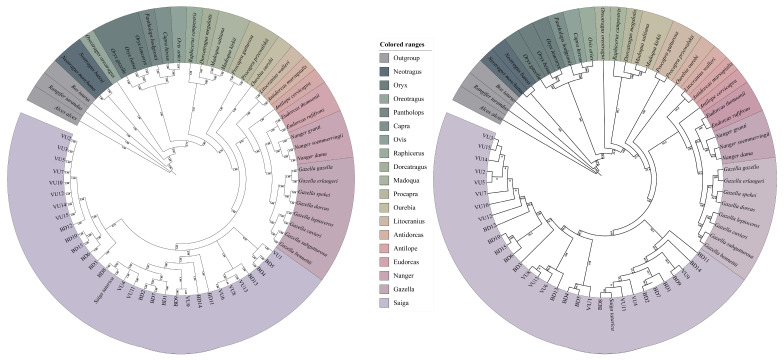
Bayesian and maximum likelihood phylogenetic trees of *Saiga tatarica* in Kazakhstan constructed from complete mitogenomes, showing congruent topologies (BD = Betpak-dala, U = Ural).

The phylogenetic trees obtained from maximum likelihood (ML) and Bayesian inference (BI) analyses exhibit broadly consistent topologies, with most major clades receiving strong support. In the BI tree, most major nodes are supported by high posterior probabilities, whereas in the ML tree, some nodes have relatively lower bootstrap support values (Figure 3). The outgroups used in this study—*Bos taurus*, *Rangifer tarandus*, and *Alces alces*—occupied stable basal positions in both phylogenetic trees and remained unchanged. The 30 samples clustered with the saiga antelope reference genome, while Procapra formed a distinct clade. *Raphicerus*, *Dorcatragus*, and *Madoqua* grouped together, whereas *Gazella*, *Nanger*, and *Eudorcas* were closely related to saiga antelope. These results collectively indicate that the phylogenetic relationships inferred in this study are highly reliable.

### 3.2. Genetic Diversity and Population Differentiation Analysis

Based on 30 complete mitochondrial genome sequences, after removing sites containing gaps or missing data, a total of 16,367 bp of valid sites were obtained for population genetic diversity analysis. In the total sample, 406 polymorphic sites were detected, including 182 singleton variable sites and 224 parsimony-informative sites, with a total of 416 mutations. A total of 15,961 conserved sites were identified, with only four sites exhibiting missing data or gaps (Table 2). At the population level, the BD population harbored 338 polymorphic sites, with *k* = 81.914, *π* = 0.00500, 13 haplotypes, and *Hd* = 0.981. In contrast, the U population exhibited 230 polymorphic sites, with *k* = 67.952, *π* = 0.00415, nine haplotypes, and *Hd* = 0.848 (Table 2).

**Figure 4:**
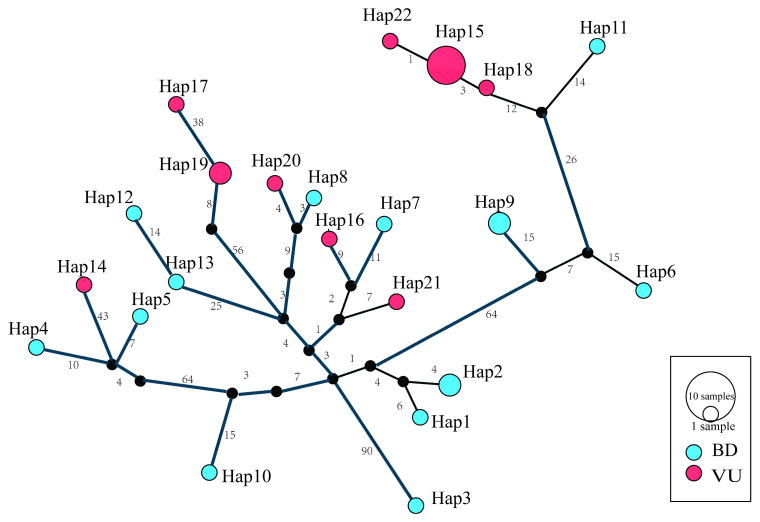
Median-joining network of 22 haplotypes in *Saiga tatarica* in Kazakhstan, constructed using PopART. Circle sizes are proportional to the number of individuals, Numbers indicate the mutational step size.

The haplotype network constructed based on TCS (Figure 4) identified a total of 22 haplotypes. Among them, Hap15 is the predominant haplotype (*N* = 6) and is found exclusively in the VU population. The BD population has 13 private haplotypes (Hap1–Hap13), with Hap2 and Hap9 each containing two individuals. The VU population has 9 private haplotypes (Hap14–Hap22), with Hap19 containing two individuals. Notably, no shared haplotypes are detected between the two populations.

**Figure 5:**
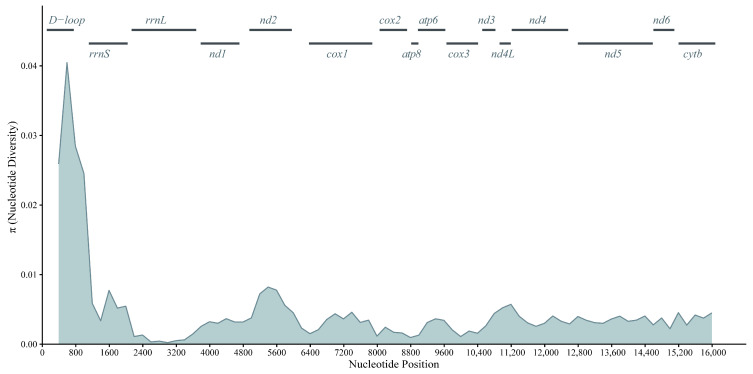
Sliding window analysis of nucleotide diversity (*π*) based on 30 complete mitogenomes.

To further characterize the patterns of genetic variation across different regions of the mitochondrial genome, we employed a sliding-window analysis (window size: 600 bp; step size: 200 bp) to assess the distribution of nucleotide diversity across the entire genome. The results show that nucleotide diversity is unevenly distributed across the genome, with higher levels of polymorphism observed in the D-loop region and the *nd2* gene (Figure 5).

### 3.3. Population Genetic Structure and Neutrality Tests

To assess the genetic structure and selection pressures between the BD and VU saiga populations, we calculated genetic differentiation indices and neutrality test statistics. Analysis of genetic differentiation between the populations showed that no fixed differences were detected between the BD and VU populations, but there were 183 private mutations in the BD population, 71 private mutations in the VU population, and 162 shared mutations. The *Fst* value was 0.12329, corresponding to an *Nm* of 3.56, indicating a moderate level of genetic differentiation between the two populations. The nucleotide divergence (*Dxy*) was 0.00522, and the net number of nucleotide differences (*Da*) was 0.00064 (Table 3).

**Figure 6:**
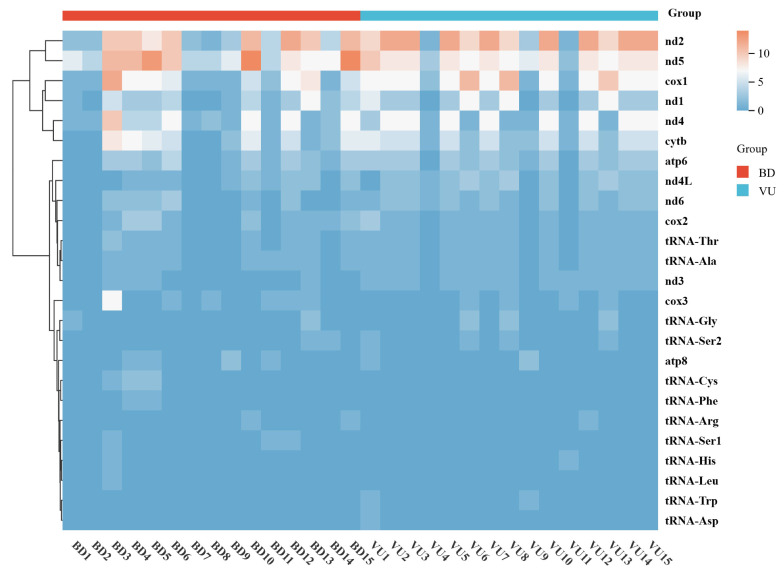
Distribution of variants across mitochondrial genes in VU and BD populations. Heatmap showing the distribution of variants across all genes (*y*-axis) and samples (*x*-axis), with color intensity representing variant count as indicated in the legend.

A heatmap constructed based on the number of SNPs in different gene regions of the mitochondrial genome reveals that SNPs are predominantly concentrated in protein coding genes such as *nd2*, *nd5*, *cox1*, *nd1*, *nd4*, and *cytb*. Among these, the *nd2*, *nd5*, and *cytb* regions exhibit relatively high levels of variation, whereas the *atp8* gene and most tRNA encoding regions harbor fewer SNPs (Figure 6) (Table S3). Analysis of synonymous and nonsynonymous mutations, as well as transitions and transversions, across 13 PCGs further supports this distribution pattern: the *nd2* and *nd5* genes exhibit the highest number of mutations, whereas *atp8* and most tRNA genes show the fewest, with transitions predominating among the mutation types (Tables S4 and S5).

In Tajima’s D test, none of the total sample (−0.84003), the BD population (−0.92389), or the VU population (−0.17421) reached statistical significance. Fu and Li’s D* and F* tests were also not significant in all populations (Table 4). However, Fu’s Fs test was significantly positive in the VU population (9.187, *p* < 0.01) and Strobeck’s test was also significant (0.001, *p* < 0.01), indicating that the number of haplotypes in the VU population was significantly lower than the neutral expectation. Fu’s Fs (2.225 and 3.882) and Strobeck’s (0.318 and 0.056) were not significant for both the BD population and the overall sample. In summary, except for the VU population, the remaining test results support the neutral evolutionary model.

## 4. Discussion

This study, based on complete mitochondrial genome data, systematically revealed the genetic diversity, matrilineal genetic structure, and functional gene variants of BD and VU populations. The results showed that the complete mitochondrial genomes of both populations were 16,376 bp in length, with an average AT content of approximately 62%. Their gene composition and codon usage patterns were generally consistent with subfamily Antilopinae and other ungulate species [21,22]. In addition, incomplete stop codons (TA/T) were detected in the *nd1*, *nd2*, *cox3*, *nd3*, and *nd4* genes, which are presumed to be converted into complete TAA stop codons through post-transcriptional polyadenylation [3]. Overall, the mitochondrial genome structures of the BD and VU populations were relatively conserved, with no obvious structural differences observed between the two populations.

The topological structures of the phylogenetic trees constructed using ML and BI based on complete mitochondrial genomes were largely consistent, indicating that the phylogenetic relationships inferred in this study are highly reliable. This study found that the *Saiga tatarica* is closely related to the genera *Gazella*, *Nanger*, and *Eudorcas*, which is consistent with previous studies [3,23]. Furthermore, population genetic diversity analysis showed that the BD and VU populations still retained relatively high mitochondrial genetic diversity. The π values for both populations fall within the range observed for complete mitochondrial genome diversity in mammals [24]. In addition to historical population factors, environmental and ecological factors may also affect mitochondrial genomic variation among different populations of saiga antelopes. The saiga antelope lives in highly seasonal grasslands, semi-deserts, and desert ecosystems, and tracks suitable climate conditions and forage resources through seasonal migrations [25]. Long-term exposure to arid habitats, a strong continental climate, food restrictions caused by ice and snow, disease outbreaks, and the pressure of energy consumption during migration may selectively affect genes related to energy metabolism, stress response, immunity, and environmental adaptation [4,26]. Therefore, the high genetic diversity of the BD population in this study may be partly related to its large historical effective population size, wide habitat range, and diverse ecological environment. In comparison, although the population of the VU group has increased significantly in recent years, its distribution area is relatively concentrated, the types of habitats are relatively simple, and it has been subject to strong local protection and management interventions for a long time [4]. While such measures have contributed to population recovery, they may also have limited individual dispersal and opportunities for random mating to some extent, potentially resulting in a reduction in matrilineal genetic diversity [27]. Moreover, habitat fragmentation caused by linear infrastructure and other human disturbances may limit individual dispersal and genetic exchange, thereby affecting the genetic structure of the population in the long run [28].

Unlike earlier studies based on mtDNA fragments, this study used the complete mitochondrial genome to obtain a higher genetic resolution. Haplotype network analysis further indicated a certain degree of mitochondrial genetic differentiation between the two populations, but the level of differentiation has not yet reached that expected under long-term complete isolation. A total of 22 haplotypes were detected in the two populations: 13 in the BD population and 9 in the VU population. No shared haplotypes were found between the two populations, indicating that the two populations have already diverged in matrilineal genetic composition. However, the BD and VU haplotypes did not form completely independent lineages in the network, and some haplotypes showed an intermingled distribution. This suggests that although recognizable matrilineal genetic differences have emerged between the two populations, complete lineage sorting has not yet occurred [29]. Compared with the results obtained by Kholodova et al. [30] based on the HV1 fragment, the high resolution of the complete mitochondrial genome helps to reveal a more detailed phylogeographic structure.

Mitochondrial protein-coding genes are primarily involved in oxidative phosphorylation, and their functional stability is critical for energy metabolism [10]. Consequently, non-synonymous mutations are typically subject to strong purifying selection, whereas synonymous mutations tend to accumulate more readily within populations [31]. The SNP analysis in this study revealed that, across all protein-coding genes, the number of synonymous mutations significantly exceeds that of non-synonymous mutations, indicating that the mitochondrial protein-coding genes of the saiga antelope are subject to strong purifying selection [32]. Although mitochondrial protein-coding genes are generally highly conserved, sliding-window analyses and SNP distribution patterns reveal that the level of variation is uneven across different genomic regions. Notably, the D-loop, *nd2*, and *nd5* regions exhibit relatively high nucleotide diversity. As the major non-coding regulatory region of the mitochondrial genome, the D-loop exhibits a relatively rapid evolutionary rate and a high mutation rate; consequently, its level of variation is typically higher than that of the coding regions, which is consistent with previous findings [33]. Unlike the high variability observed in the D-loop, which is a common feature of non-coding regions, this study also found that certain protein-coding gene regions have similarly accumulated substantial genetic variation. Notably, *nd2* and *nd5* harbor relatively large numbers of SNPs. To account for the confounding effect of gene length on SNP counts, we further calculated SNP density (SNPs per kilobase) for each mitochondrial protein-coding gene. It shows that *nd2* has the highest SNP density in both groups, indicating that its high number of SNPs is not solely due to the length of the gene, but also reflects the high degree of variation in the gene. This pattern is also consistent with findings in *S. t. tatarica*. Based on complete mitochondrial genome analysis, Ulyanov et al. [2] reported that *nd2* and *nd5* contained more variable sites than most other mitochondrial protein-coding genes. Combined with our SNP density analysis, these findings suggest that nd2 is indeed a hotspot for mitochondrial mutations, whereas the higher SNP count in nd5 is likely partly explained by its longer coding sequence.

Similar phenomena have also been reported in studies of mitochondrial genomes from other animals. For example, research on the mitochondrial genome of the genus Camelus has revealed that *nd*-class genes exhibit a relatively high evolutionary rate, and that multiple sites potentially subject to selective pressure are present in *nd5* [31]. Similarly, a study on the mitochondrial genome of the genus Pica revealed that the *nd4*, *nd5*, and *nd6* regions also exhibit high levels of genetic differentiation, and the researchers suggest that variations in these genes may be associated with the regulation of energy metabolism and environmental adaptation [34]. It is worth noting that the BD and VU populations differ in their geographic distribution, habitat types, and conservation management practices, factors that may influence the formation of their genetic structure [4]. Therefore, the relatively high levels of variation observed in nd-type genes, particularly *nd2*, may be linked to the population’s evolutionary history and environmental adaptation. However, mitochondrial genome data alone are insufficient to substantiate their adaptive significance; further research is needed, incorporating nuclear genome data, analyses of environmental factors, and functional experiments.

The neutrality test results showed that all statistical parameters of the BD group did not deviate significantly from the neutral model, indicating that the group has not experienced significant population expansion, bottleneck, or strong selection pressure in the recent past [35]. In comparison, the results of Fu’s Fs and Strobeck’s S tests in the VU group deviated significantly from the neutral expectation, indicating that the number of their haplotypes was lower than the neutral expectation [36]. This deviation may be related to factors such as fluctuations in the effective population size, genetic drift, and natural selection or population structure in their historical populations [37]. In comparison, the BD group may maintain a more stable haplotype structure due to its larger historical effective population size or more abundant gene flow [4].

Several limitations should be acknowledged in this study. First, although the samples of the BD and VU groups were collected randomly, the relatively small sample size may have limited the study’s ability to comprehensively capture the spatial genetic variation and potential population substructures of the saiga antelope over a larger geographical area. In addition, this study did not assess kinship among individuals based on nuclear markers, nor did we screen for nuclear mitochondrial DNA segments (NUMTs); therefore, we cannot exclude the possibility of close relatedness or contamination by NUMTs in mitochondrial assemblies. Although we validated the assembly results by comparison with the reference genome and verification of gene annotation completeness, the potential impact of NUMTs cannot be fully excluded. Future research should expand the sample size and geographical sampling range, and combine nuclear genome data, long-read sequencing technology, and specialized NUMTs detection methods to more accurately assess the genetic diversity, kinship, population structure, and mitochondrial variation characteristics of Saiga antelopes.

## 5. Conclusions

Overall, the BD and VU groups have shown certain differentiation in maternal inheritance and maintained a high level of genetic diversity. No fixed difference sites were detected between the two groups, indicating that their degree of differentiation is still relatively limited, which may reflect a recent history of differentiation or ongoing maternal gene flow. The mitochondrial genome variants are mainly concentrated in the D-loop and some *nd*-class gene regions, among which *nd5* exhibits a high level of variation, suggesting that it may record genetic information related to the historical evolution of populations or environmental adaptation. This study provides a fundamental basis for further combining nuclear genome data to analyze the mechanisms of population differentiation, adaptive evolution, and conservation unit delineation in the saiga antelope.

## Figures and Tables

**Table 1:** Mitochondrial gene annotation (PCGs, tRNAs, rRNAs) of *Saiga tatarica* in Kazakhstan.

Gene	Type (bp)	Start (bp)	End (bp)	Length (bp)	Start Codon	Termination Codon	Anticodon	Strand	Gap or Overlap
*tRNA-Phe*	tRNA	940	1007	68			GAA	+	0
*rrnS*	rRNA	1008	1962	955				+	0
*tRNA-Val*	tRNA	1963	2029	67			UAC	+	0
*rrnL*	rRNA	2030	3601	1572				+	0
*tRNA-Leu1*	tRNA	3602	3676	75			UAA	+	0
*nd1*	CDS	3679	4634	956	ATG	TA		+	2
*tRNA-Ile*	tRNA	4635	4703	69			GAU	+	0
*tRNA-Gln*	tRNA	4701	4772	72			UUG	−	−3
*tRNA-Met*	tRNA	4775	4843	69			CAU	+	2
*nd2*	CDS	4844	5885	1042	ATA	T		+	0
*tRNA-Trp*	tRNA	5886	5952	67			UCA	+	0
*tRNA-Ala*	tRNA	5954	6022	69			UGC	−	1
*tRNA-Asn*	tRNA	6024	6096	73			GUU	−	1
*tRNA-Cys*	tRNA	6129	6195	67			GCA	−	32
*tRNA-Tyr*	tRNA	6196	6263	68			GUA	−	0
*cox1*	CDS	6265	7809	1545	ATG	TAA		+	1
*tRNA-Ser1*	tRNA	7807	7875	69			UGA	−	−3
*tRNA-Asp*	tRNA	7884	7951	68			GUC	+	8
*cox2*	CDS	7953	8636	684	ATG	TAA		+	1
*tRNA-Lys*	tRNA	8640	8707	68			UUU	+	3
*atp8*	CDS	8709	8909	201	ATG	TAA		+	1
*atp6*	CDS	8870	9550	681	ATG	TAA		+	−40
*cox3*	CDS	9550	10,333	784	ATG	T		+	−1
*tRNA-Gly*	tRNA	10,334	10,402	69			UCC	+	0
*nd3*	CDS	10,403	10,748	346	ATA	T		+	0
*tRNA-Arg*	tRNA	10,750	10,818	69			UCG	+	1
*nd4L*	CDS	10,819	11,115	297	ATG	TAA		+	0
*nd4*	CDS	11,109	12,486	1378	ATG	T		+	−7
*tRNA-His*	tRNA	12,487	12,557	71			GUG	+	0
*tRNA-Ser2*	tRNA	12,558	12,617	60			GCU	+	0
*tRNA-Leu2*	tRNA	12,619	12,688	70			UAG	+	1
*nd5*	CDS	12,689	14,509	1821	ATA	TAA		+	0
*nd6*	CDS	14,493	15,020	528	ATG	TAA		−	−17
*tRNA-Glu*	tRNA	15,021	15,089	69			UUC	−	0
*cytb*	CDS	15,094	16,236	1143	ATG	TAA		+	4
*tRNA-Thr*	tRNA	16,237	16,306	70			UGU	+	0
*tRNA-Pro*	tRNA	16,306	16,371	66			UGG	−	−1

Positive numbers in the “Gap or overlap” column indicate intergenic sequence length, while negative numbers indicate the length of overlapping regions between adjacent genes. The forward strands are marked as “+” and the reverse strands as “−”. “T” and “TA” denote incomplete stop codons.

**Table 2:** Mitochondrial genome genetic diversity parameters of VU and BD populations.

Population	*N*	*H*	*Hd*	Sequence Length (bp)	*S*	*Eta*	*k*	*π*
All samples	30	22	0.96	16,367	406	416	80.384	0.00491
Population BD	15	13	0.98	16,367	338	345	81.914	0.005
Population VU	15	9	0.85	16,367	230	233	67.952	0.00415

*H*, Number of haplotypes; *S*, variable sites; *Eta*, total mutations; *k*, Average number of nucleotide differences; *π*, Nucleotide diversity.

**Table 3:** Mitochondrial genetic differentiation parameters between VU and BD populations.

Population Comparison	Fixed Differences	BD-Specific Mutations	VU-Specific Mutations	Shared Mutations	Average Number of Nucleotide Substitutions	*Dxy*	*Da*	*Fst*
BD vs. VU	0	183	71	162	85.471	0.0052	0.0006	0.1233

*Dxy*, average nucleotide substitutions per site between populations; *Da*, net nucleotide substitutions per site.

**Table 4:** Population Genetic Diversity and Neutrality Test Statistics.

Population	θw (per Site)	Tajima’s D	Fu’s Fs	Fu and Li’s D*	Fu and Li’s F*	Strobeck’s S
All samples	0.0063	−0.8400	3.8820	−1.4599	−1.3966	0.0560
Population BD	0.0064	−0.9240	2.2250	−1.1399	−1.2450	0.3180
Population VU	0.0043	−0.1740	9.1870	−0.5530	−0.5151	0.001

*θw*, Watterson’s estimator of population mutation rate per nucleotide site; *π*, nucleotide diversity; *D* and *F* represent Fu and Li’s neutrality test statistics; the asterisk “*” is part of the notation for Fu and Li’s *D* and *F* statistics and does not indicate statistical significance.

## Data Availability

The data that support the findings of this study are available from the corresponding author upon reasonable request.

## Supplementary Materials

The following supporting information can be downloaded at https://www.mdpi.com/article/10.3390/ani16142256/s1, Table S1: Species included in the phylogenetic analyses and their GenBank accession numbers; Table S2: Quality statistics of filtered reads; Table S3: SNP densities of mitochondrial protein-coding genes in BD and VU populations; Table S4: The number of synonymous and nonsynonymous sites in the 13 PCGs; Table S5: The number of transition/transversion mutations in the 13 PCGs and 22 tRNAs.

## References

[1] Wang S. (2019). Study on Diet Habit and Daytime Activity of Semi-Dispersed *Saiga tatarica* in Gansu Endangered Animal Protection Center. Master’s Thesis.

[2] Ulyanov V., Beishova I., Yanin K., Ulyanova T., Ginayatov N., Kovalchuk A., Shamshidin A., Kushaliyev K., Tleulenov Z., Karimov A. (2026). Comparative assessment of interindividual variability of the mitochondrial and nuclear genomes in *Saiga tatarica tatarica*. Front. Anim. Sci..

[3] Ding X., Wu J., Xiao H., Wang Z., Liu Q., Liu X., Jin K., Zheng D. (2017). Complete mitochondrial genome of *Saiga tatarica* (Ruminantia; Pecora; Bovidae) isolate Wuwei in China. Mitochondrial DNA B Resour..

[4] Serikbayeva A.T., Akimzhanov D.S., Iskakova Z.A., Karagoishin Z., Akoyev M.T., Dauletaliyev T.N. (2023). Baitanayev OA: Saiga (*Saiga tatarica*) conservation strategy in Kazakhstan. Braz. J. Biol..

[5] Meng Z. (2011). Explanations on the CITES and the Domestic Policies in relation to Saiga (*Saiga tatarica*). Mod. Chin. Med..

[6] Kashinina N.V., Lushchekina A.A., Sorokin P.A., Tarasyan K.K., Kholodova M.V. (2023). The modern state of the European saiga population (*Saiga tatarica tatarica*): mtDNA, DRB3 MHC gene, and microsatellite diversity. Integr. Zool..

[7] Karimova T.Y., Lushchekina A.A., Neronov V.M. (2021). Saiga Populations of Russia and Kazakhstan: Current Status and Retrospective Analysis of Some Biological Parameters. Arid. Ecosyst..

[8] Shaw R.E., Farquharson K.A., Bruford M.W., Coates D.J., Elliott C.P., Mergeay J., Ottewell K.M., Segelbacher G., Hoban S., Hvilsom C. (2025). Global meta-analysis shows action is needed to halt genetic diversity loss. Nature.

[9] Rey-Iglesia A., Hjort J., Silva T.L., Buuveibaatar B., Dalannast M., Ulziisaikhan T., Chimeddorj B., Espregueira-Themudo G. (2022). CamposPF: Genetic diversity of the endangered Mongolian saiga antelope *Saiga tatarica* mongolica (Artiodactyla: Bovidae) provides insights into conservation. Biol. J. Linn. Soc..

[10] Campos P.F., Kristensen T., Orlando L., Sher A., Kholodova M.V., Götherström A., Hofreiter M., Drucker D.G., Kosintsev P., Tikhonov A. (2010). Ancient DNA sequences point to a large loss of mitochondrial genetic diversity in the saiga antelope (*Saiga tatarica*) since the Pleistocene. Mol. Ecol..

[11] Wen B. (2025). Study on Population Structure and Genetic Variation of Energy Metabolism-Related Genes in Mongolian Cattle and Their Hybrid Strains. Master’s Thesis.

[12] Liu N., Wang H., Fang L., Zhang Y. (2023). Mitogenome of the Doleschallia bisaltide and Phylogenetic Analysis of Nymphalinae (Lepidoptera, Nymphalidae). Diversity.

[13] Letunic I., Bork P. (2024). Interactive Tree of Life (iTOL) v6: Recent updates to the phylogenetic tree display and annotation tool. Nucleic Acids Res..

[14] Katoh K., Toh H. (2010). Parallelization of the MAFFT multiple sequence alignment program. Bioinformatics.

[15] Koichiro T., Glen S., Sudhir K. (2021). MEGA11: Molecular Evolutionary Genetics Analysis Version 11. Mol. Biol. Evol..

[16] Rozas J., Ferrer-Mata A., Sánchez-DelBarrio J.C., Guirao-Rico S., Librado P., Ramos-Onsins S.E., Sánchez-Gracia A. (2017). DnaSP 6: DNA Sequence Polymorphism Analysis of Large Data Sets. Mol. Biol. Evol..

[17] Leigh J.W., Bryant D. (2015). Popart: Full-feature software for haplotype network construction. Methods Ecol. Evol..

[18] Minh B.Q., Schmidt H., Chernomor O., Schrempf D., Woodhams M., von Haeseler A., Lanfear R. (2020). IQ-TREE 2: New Models and Efficient Methods for Phylogenetic Inference in the Genomic Era. Mol. Biol. Evol..

[19] Ronquist F., Teslenko M., van der Mark P., Ayres D.L., Darling A., Höhna S., Larget B., Liu L., Suchard M.A., Huelsenbeck J.P. (2012). MrBayes 3.2: Efficient Bayesian phylogenetic inference and model choice across a large model space. Syst. Biol..

[20] Letunic I., Bork P. (2007). Interactive Tree Of Life (iTOL): An online tool for phylogenetic tree display and annotation. Bioinformatics.

[21] Ghassemi-Khademi T. (2017). Evaluation of phylogenetic relationships of Antilopini and Oreotragini tribes (Bovidae: Artiodactyla) based on complete mitochondrial genomes. J. Wildl. Biodivers..

[22] Guo X., Pei J., Zhou Y., Bao P., Liu J., Hanzhong J.I., Xiaoyun W.U., Ding X., Yan P., Zhao S. (2017). Characterization of the Complete Mitochondrial Genome of Przewalski’s Gazelle *Procapra przewalskii* in Bovidae Family of Artiodactyla. Agric. Biotechnol..

[23] Cernohorska H., Kubickova S., Kopecna O., Vozdova M., Matthee C.A., Robinson T.J., Rubes J. (2015). Nanger, Eudorcas, Gazella, and Antilope form a well-supported chromosomal clade within Antilopini (Bovidae, Cetartiodactyla). Chromosoma.

[24] Singh B., Kumar A., Uniyal V.P., Gupta S.K. (2021). Phylogeography and population genetic structure of red muntjacs: Evidence of enigmatic *Himalayan red muntjac* from India. BMC Ecol. Evol..

[25] Mullineaux S.T., McKinley J.M., Marks N.J., Doherty R., Scantlebury D.M. (2024). A nose for trouble: Ecotoxicological implications for climate change and disease in Saiga antelope (*S. t. tatarica*). Environ. Geochem. Health.

[26] Chebii V.J., Mpolya E.A., Muchadeyi F.C., Domelevo Entfellner J.-B. (2021). Genomics of Adaptations in Ungulates. Animals.

[27] Farquharson K.A., Hogg C.J., Grueber C.E. (2021). Offspring survival changes over generations of captive breeding. Nat. Commun..

[28] Bizhanova N., Grachev A., Rametov N., Baidavletov Y., Saparbayev S., Bespalov M., Bespalov S., Kumayeva I., Toishibekov Y., Khamchukova A. (2025). Railway and Road Infrastructure in Saiga Antelope Range in Kazakhstan. Diversity.

[29] Srikulnath K., Ariyaraphong N., Singchat W., Panthum T., Lisachov A., Ahmad S.F., Han K., Muangmai N., Duengkae P. (2023). Asian Elephant Evolutionary Relationships: New Perspectives from Mitochondrial D-Loop Haplotype Diversity. Sustainability.

[30] Kholodova M.V., Milner-Gulland E.J., Easton A.J., Amgalan L., Arylov I., Bekenov A. (2006). Mitochondrial DNA variation and population structure of the Critically Endangered saiga antelope *Saiga tatarica*. Oryx.

[31] Mohandesan E., Fitak R.R., Corander J., Yadamsuren A., Chuluunbat B., Abdelhadi O., Raziq A., Nagy P., Stalder G., Walzer C. (2017). Mitogenome Sequencing in the Genus Camelus Reveals Evidence for Purifying Selection and Long-term Divergence between Wild and Domestic Bactrian Camels. Sci. Rep..

[32] Kadam P.S., Yang Z., Lu Y., Zhu H., Atiyas Y., Shah N., Fisher S., Nordgren E., Kim J., Issadore D. (2024). Single-mitochondrion sequencing uncovers distinct mutational patterns and heteroplasmy landscape in mouse astrocytes and neurons. BMC Biol..

[33] Zorkóczy O.K., Wagenhoffer Z., Lehotzky P., Pádár Z., Zenke P. (2024). Mitochondrial Control Region Database of Hungarian Fallow Deer (*Dama dama*) Populations for Forensic Use. Animals.

[34] Kryukov A.P., Kryukov K.A., Collier K., Fang B., Edwards S.V. (2024). Mitogenomics clarifies the position of the Nearctic magpies (*Pica hudsonia* and *Pica nuttalli*) within the Holarctic magpie radiation. Curr. Zool..

[35] Badoni P., Rauthan J.V. (2026). S: Mitochondrial marker-based insights into the genetic diversity and population connectivity of the vulnerable (IUCN Red List) snow trout (*Schizothorax plagiostomus*) from the Tons River, Western Himalaya. Front. Aquac..

[36] Ullah I., Sattar S., Ali I., Farid A., Ullah A., Eid R.A., Samir A.Z.M., Alaa Eldeen M., Ahmed I., Ullah I. (2023). Molecular Epidemiology of Cystic Echinococcosis in Rural Baluchistan, Pakistan: A Cross-Sectional Study. Pathogens.

[37] Elgvin T.O., Trier C.N., Tørresen O.K., Hagen I.J., Lien S., Nederbragt A.J., Ravinet M., Jensen H., Sætre G.P. (2017). The genomic mosaicism of hybrid speciation. Sci. Adv..

