# Supplementary material for: Mitochondrial Genomic Characteristics and Maternal Genetic Differentiation of Different Geographical Populations of Saiga tatarica in Kazakhstan
Source: Animals (Basel). 2026 Jul 21;16(14):2256. doi: 10.3390/ani16142256 (PMC13405350; doi:10.3390/ani16142256)
Supplement: Supplementary file 1 [file animals-16-02256-s001.zip › animals-4423826-supplementary.pdf]

Mitochondrial Genomic Characteristics and Maternal Genetic Differentiation of Different Geographical Populations of *Saiga tatarica* in Kazakhstan

Yue Pang [1,†], Zhumanov Kairat Toksanbaevich [1,2,†], Siyuan Wang [1], Nurpeisova Ainur Sultanovna [3], Bakirov Nurbol Zhumagadyrovish [2], Smagulov Darkhan Bakytbekovich [4] and Wurelihazi Hazihan [1,*]

[1] College of Animal Science and Technology, Shihezi University, Shihezi 832000, China; pangyue515@163.com (Y.P.); kairat_nur85@mail.ru (Z.K.T.); wangsiyuanme@126.com (S.W.)
[2] Faculty of Veterinary Medicine, Kazakh National Agrarian Research University, Almaty 050010, Kazakhstan; nurbol979@mail.ru
[3] Kazakh Scientific Research Veterinary Institute, Almaty 050010, Kazakhstan; nurai1005@gmail.com
[4] Faculty of Agriculture and Veterinary Medicine, West Kazakhstan Innovation and Technology University, Uralsk 090009, Kazakhstan; dark.smagul@gmail.com
* Correspondence: wurelihazi@shzu.edu.cn
† These authors contributed equally to this work.

Table S1. Species included in the phylogenetic analyses and their GenBank accession numbers.

No.	Species	Genus	GenBank accession No.
1	*Saiga tatarica*	*Saiga*	NC_020746.1
2	*Gazella bennettii*	*Gazella*	NC_020703.1
3	*Gazella cuvieri*	*Gazella*	NC_020704.1
4	*Gazella dorcas*	*Gazella*	NC_020705.1
5	*Gazella erlangeri*	*Gazella*	NC_020706.1
6	*Gazella gazella*	*Gazella*	NC_020707.1
7	*Gazella leptoceros*	*Gazella*	NC_020708.1
8	*Gazella spekei*	*Gazella*	NC_020709.1
9	*Gazella subgutturosa*	*Gazella*	NC_020710.1
10	*Nanger dama*	*Nanger*	NC_020724.1
11	*Nanger granti*	*Nanger*	NC_020725.1
12	*Nanger soemmerringii*	*Nanger*	NC_020726.1
13	*Eudorcas rufifrons*	*Eudorcas*	NC_020702.1
14	*Eudorcas thomsonii*	*Eudorcas*	NC_039669.1
15	*Antilope cervicapra*	*Antilope*	NC_012098.1
16	*Antidorcas marsupialis*	*Antidorcas*	NC_020678.1
17	*Litocranius walleri*	*Litocranius*	NC_020716.1
18	*Ourebia ourebi*	*Ourebia*	NC_020733.1
19	*Procapra gutturosa*	*Procapra*	NC_020738.1
20	*Procapra przewalskii*	*Procapra*	NC_014875.1
21	*Madoqua saltiana*	*Madoqua*	NC_020718.1
22	*Madoqua kirkii*	*Madoqua*	NC_020717.1
23	*Dorcatragus megalotis*	*Dorcatragus*	NC_020701.1
24	*Raphicerus campestris*	*Raphicerus*	NC_020741.1
25	*Oreotragus oreotragus*	*Oreotragus*	NC_020731.1
26	*Ovis aries*	*Ovis*	NC_001941.1
27	*Capra hircus*	*Capra*	NC_005044.2
28	*Pantholops hodgsonii*	*Pantholops*	NC_007441.1
29	*Oryx leucoryx*	*Oryx*	NC_020732.1
30	*Oryx beisa*	*Oryx*	NC_020793.1
31	*Oryx gazella*	*Oryx*	NC_016422.1
32	*Neotragus batesi*	*Neotragus*	NC_020727.1
33	*Neotragus moschatus*	*Neotragus*	NC_020728.1
34	*Bos taurus*	*Bos*	NC_006853.1
35	*Rangifer tarandus*	*Rangifer*	NC_007703.1
36	*Alces alces*	*Alces*	NC_020677.1

Table S2. Quality statistics of filtered reads.

Sample	Raw Base(G)	Clean Base(G)	Error(%)	Q20(%)	Q30(%)
BD1	6.43	6.41	0.01	99.53	97.85
BD2	6.27	6.24	0.01	99.56	98.04
BD3	6.08	6.06	0.01	99.53	97.93
BD4	6.48	6.46	0.01	99.56	98.03
BD5	6.16	6.14	0.01	99.54	97.92
BD6	6.12	6.1	0.01	99.55	97.96
BD7	5.93	5.91	0.01	99.53	97.96
BD8	6.24	6.21	0.01	99.55	98.01
BD9	6.49	6.46	0.01	99.54	98.00
BD10	6.09	6.07	0.01	99.55	98.00
BD11	6.35	6.33	0.01	99.56	98.03
BD12	6.66	6.64	0.01	99.54	97.93
BD13	5.71	5.68	0.01	99.33	97.14
BD14	6.49	6.46	0.01	99.38	97.29
BD15	6.68	6.62	0.01	99.39	97.24
VU1	5.58	5.56	0.01	99.52	97.73
VU2	6.48	6.45	0.01	99.53	97.98
VU3	6.17	6.15	0.01	99.55	97.92
VU4	6.41	6.38	0.01	99.54	97.98
VU5	6.46	6.44	0.01	99.54	97.96
VU6	6.27	6.25	0.01	99.57	98.01
VU7	6.44	6.43	0.01	99.55	97.94
VU8	6.22	6.20	0.01	99.55	98.03
VU9	5.91	5.89	0.01	99.57	98.06
VU10	6.40	6.38	0.01	99.55	97.97
VU11	6.14	6.12	0.01	99.56	98.04
VU12	6.43	6.41	0.01	99.55	97.98
VU13	6.21	6.19	0.01	99.55	97.96
VU14	6.37	6.35	0.01	99.57	98.07
VU15	6.10	6.08	0.01	99.54	97.94

Table S3. SNP densities of mitochondrial protein-coding genes in BD and VU populations.

Gene	Length (bp)	BD population SNPs	BD density (SNPs/kb)	VU population SNPs	VU density (SNPs/kb)
cox1	1545	65	42.071	100	64.725
cox2	684	15	21.930	14	20.468
cox3	784	12	15.306	4	5.102
cytb	1143	49	42.870	56	48.994
nd1	956	38	39.749	51	53.347
nd2	1042	100	95.969	137	131.478
nd3	346	4	11.561	13	37.572
nd4	1378	56	40.639	65	47.170
nd4L	297	13	43.771	26	87.542
nd5	1821	123	67.545	106	58.210
nd6	528	13	24.621	21	39.773
atp6	681	26	38.179	34	49.927
atp8	201	5	24.876	3	14.925

Table S4. The number of synonymous and nonsynonymous sites in the 13 PCGs.

Gene	BD synonymous	BD nonsynonymous	VU synonymous	VU nonsynonymous
cox1	49	16	85	15
cox2	15	0	14	0
cox3	12	0	4	0
cytb	44	5	55	1
nd1	37	1	48	3
nd2	86	14	119	18
nd3	4	0	12	1
nd4	50	6	57	8
nd4L	11	2	26	0
nd5	85	38	70	36
nd6	13	0	21	0
atp6	22	4	25	9
atp8	2	3	1	2
Total	430	89	537	93

Table S5. The number of transition/transversion mutations in the 13 PCGs and 22 tRNAs.

Gene type	Gene	BD transition	BD transversion	VU transition	VU transversion
PCG	cox1	63	2	94	6
PCG	cox2	15	0	14	0
PCG	cox3	12	0	4	0
PCG	cytb	47	2	56	0
PCG	nad1	38	0	51	0
PCG	nad2	100	0	136	1
PCG	nad3	4	0	13	0
PCG	nad4	56	0	65	0
PCG	nd4L	13	0	26	0
PCG	nd5	121	2	103	3
PCG	nd6	13	0	18	3
PCG	atp6	26	0	34	0
PCG	atp8	5	0	3	0
tRNA	tRNA-Pro	0	0	0	0
tRNA	tRNA-Thr	9	0	12	0
tRNA	tRNA-Glu	0	0	0	0
tRNA	tRNA-Leu1	0	0	0	0
tRNA	tRNA-Ser1	2	1	0	0
tRNA	tRNA-His	1	0	0	1
tRNA	tRNA-Arg	2	0	1	0
tRNA	tRNA-Gly	3	0	6	0
tRNA	tRNA-Lys	0	0	0	0
tRNA	tRNA-Asp	0	0	1	0
tRNA	tRNA-Ser2	0	2	1	3
tRNA	tRNA-Tyr	0	0	0	0
tRNA	tRNA-Cys	5	0	0	0
tRNA	tRNA-Asn	0	0	0	0
tRNA	tRNA-Ala	9	0	12	0
tRNA	tRNA-Trp	0	0	2	0
tRNA	tRNA-Met	0	0	0	0
tRNA	tRNA-Gln	0	0	0	0
tRNA	tRNA-Ile	0	0	0	0
tRNA	tRNA-Leu2	1	0	0	0
tRNA	tRNA-Val	0	0	0	0
tRNA	tRNA-Phe	2	0	0	0
Total		547	9	652	17
